# Sexual Harassment Experiences Across the Academic Medicine Hierarchy

**DOI:** 10.7759/cureus.13508

**Published:** 2021-02-23

**Authors:** Chu J Hsiao, Neeka N Akhavan, Naykky Singh Ospina, Kruti J Yagnik, Patrick Neilan, Paulette Hahn, Zareen Zaidi

**Affiliations:** 1 Division of Anthropology, University of Florida, Gainesville, USA; 2 Division of General Internal Medicine, University of Florida, Gainesville, USA; 3 Division of Endocrinology, University of Florida, Gainesville, USA; 4 Division of Infectious Diseases and Geographic Medicine, UT Southwestern Medical Center, Dallas, USA; 5 Division of Pulmonary and Critical Care Medicine, University of Vermont, Burlington, USA; 6 Division of Rheumatology, University of Florida, Gainesville, USA

**Keywords:** culture of medicine, sexual harassment, medical education, medical students, residents, faculty

## Abstract

Background

Current estimates of sexual harassment across the academic hierarchy are subject to recall bias and have limited comparability between studies due to inconsistent time frames queried for each stage of training. No studies have surveyed medical students, residents/fellows, and faculty collectively and many studies exclude a wide range of sexual harassment behaviors. We assessed the incidence of sexual harassment across the different stages of academic medicine over the same time frame and within the same institutional culture.

Methodology

Medical students, residents/fellows, and faculty at the same academic medical campus completed a prospective online study of sexual harassment experiences in 2018. We used a tool that comprehensively assessed sexual harassment behaviors and asked about the perpetrators. Pearson’s chi-square and Fisher’s exact tests (for cell counts <5) were used to compare responses by academic status and gender. Participants were also asked to suggest ways to improve knowledge about university/hospital policies, support services, and reporting process on sexual harassment.

Results

One-third of 515 respondents (18% of invitations) reported experiencing sexual harassment in 2018. Overall, 52% of medical students, 31% of residents/fellows, and 25% of faculty respondents experienced sexual harassment. Of these, 46% of women and 19% of men reported sexual harassment experiences. The most common experiences across all levels of academic hierarchy were offensive and sexually suggestive comments or jokes and offensive and intrusive questions about one’s private life or physical appearance. The most common perpetrators were “student, intern, resident, or fellow,” followed by “patient or patient’s family member.” To improve knowledge about the policies and services regarding sexual harassment, participants suggested facilitating easy access to resources, increasing awareness, assuring confidentiality, protecting against retaliation, and continued education and reminders about the topic.

Conclusions

Sexual harassment may be more prevalent than the literature suggests and incidence tends to decrease with increasing academic hierarchy. Harassment can often be subtle and can pass under the radar.

## Introduction

Sexual harassment is a problem in academic medicine, affecting not only students and trainees but also medical faculty. Today, there are more women in leadership positions in academic medicine than ever before in the history of United States; despite this accomplishment, sexual harassment and assault on women continue to occur at all levels [1]. Sexual harassment encompasses a spectrum, including generalized sexist remarks and crude behavior, unwelcome verbal or physical sexual advances, and sexual coercion [2]. The hidden curriculum of the medical training process socializes trainees to behave appropriately within the hierarchy of academic medicine. Environments that fortify power dynamics are more likely to foster and sustain sexual harassment [2]. However, most studies capture experiences across the academic medicine hierarchy through disjointed, cross-institutional snapshots. Estimates suggest 33.1% of medical students [3], 36.2% of residents [3], and 30.4% of younger faculty [4] experience sexual harassment. It remains unclear how recall bias and differences in timeframes queried impact these figures.

According to the 2018 National Academies of Sciences, Engineering, and Medicine (NASEM) report, female medical students are 220% more likely than students from other fields to experience sexual harassment. Almost half of female medical students report experiencing some form of sexual harassment [2]. Regarding residents, a meta-analysis by Fnais et al. reported a pooled prevalence of gender discrimination of 66.6% [3] According to the NASEM report, 70% of female faculty reported gender-based discrimination, with 48% of female physicians reporting sexist comments and 30% reporting experiencing severe harassment (compared to 3% of male colleagues) [2]. In a 2003 meta-analysis examining prevalence of sexual harassment, Ilies et al. demonstrated that 58% of female academic faculty and staff experienced sexual harassment. In this study, the prevalence of sexual harassment was examined in the private, academic, military, and government sectors. The academic workplace was found to have the second highest prevalence of sexual harassment after the military (69%) [5].

These findings suggest that environments that fortify power dynamics demonstrate higher rates of sexual harassment. RTI International, a private research company, was contracted by NASEM to conduct campus climate interviews regarding sexual harassment. These interviews revealed that settings such as medical residencies were believed to be breeding grounds for sexual harassment. Multiple respondents suggested that sexual harassment was viewed as part of the continuum of abusive and difficult conditions expected to be endured by trainees [2].

To date, there has not been an institution-wide study evaluating the experiences of sexual harassment and assault among students, post-graduate trainees, faculty, and staff. This is the first report to assess and compare experiences between these groups. The StandPoint^TM^ and Graduating Questionnaire (GQ) surveys are administered by the Association of American Medical Colleges (AAMC) to faculty and medical students, respectively, to assess workplace and education climate. Although these surveys address sexual harassment to some extent, they do not provide a comprehensive evaluation. Although there have been many studies separately examining sexual harassment experiences within a specific tier of the academic medicine hierarchy, there have been none that have taken a cross-section of experiences among all ranks. We aimed to examine the incidence of sexual harassment across the different stages of the academic medicine hierarchy and within the same timeframe and institutional culture. Therefore, we conducted a survey to quantify and understand the experiences of sexual harassment and assault across the different stages among medical students, post-graduate trainees, faculty, and staff over the same timeframe at the University of Florida College of Medicine/University of Florida Health (UFCOM, UF Health). The objective of our study was to report on the prevalence of sexual harassment and assault within our institution, bring awareness to the medical community, and ultimately work toward solutions to improve the climate at our institution and beyond.

## Materials and methods

In this cross-sectional study, all medical students (540), residents/fellows (870), and faculty (1447) at the UFCOM/UF Health were invited to complete an online survey between April and May 2019. Two reminders were sent after the initial invitation email. The survey was adapted from a comprehensive tool used by the Australian Human Rights Commission to assess sexual harassment on university campuses and is available through the link in the reference provided [6]. While the tool is not a validated tool, it was chosen for its comprehensive assessment of sexual harassment behaviors. Some questions were omitted from the original survey as they were not applicable to our participants. Participants were asked to identify experiences of sexual harassment behaviors in 2018 and to classify the perpetrator(s) of the most recent episode. Additionally, they were asked to describe ways in which the UFCOM/UF Health could help ensure knowledge of university/hospital policies, support services, and reporting processes on sexual harassment. To protect anonymity, we did not collect potentially identifying demographics (i.e., race). We used Pearson’s chi-square and Fisher’s exact tests (for cell counts <5) to compare categorical responses by status and gender. All statistical analyses were conducted in R (Vienna, Austria). Two-sided P-values of <0.05 were considered statistically significant. This study was approved by the University of Florida Institutional Review Board under IRB201801056.

## Results

Of the 2,857 invitations, 145 medical students (MS; 27.0% response rate), 100 residents/fellows (RF; 11.5%), and 270 faculty (F; 19.0%) responded, totaling 515 respondents (18.0%). There were no differences in gender by status (59.3% MS, 56.0% RF, 50.0% F identified as female; P = 0.18).

One-third of respondents reported sexual harassment, with the proportion of respondents decreasing with increasing academic position (51.7% MS, 31.0% RF, 24.8% F; P < 0.001). The most common experiences across all levels of academic medicine were offensive and sexually suggestive comments or jokes (29.0% MS, 19.0% RF, 11.5% F; P < 0.001), followed by offensive and intrusive questions about one’s private life or physical appearance (23.4% MS, 15.0% RF, 5.9% F; P < 0.001). Notably, requests or pressure for sex or other sexual acts was only reported by medical students (0.4% MS, 0.0% RF, 0.0% F, P < 0.001). There was a strong gender bias where women (46.2%) experienced more sexual harassment than men (19.4%; P < 0.001), a pattern observed across all listed behaviors (Table 1).

**Table 1:** Experiences of sexual harassment by training status and sex. a: Totals add up to more than 100% because respondents indicated all that applied to them

In-person experiences	All (n = 515)	By training status, No. (%) ^a^				By sex, No. (%) ^a^		
		Medical students (n = 145)	Residents fellows (n = 100)	Faculty (n = 270)	P-Value	Women (n = 277)	Men (n = 232)	P-Value
Unwelcome touching, hugging, cornering, or kissing	49 (9.5)	18 (12.4)	10 (10.0)	21 (7.8)	0.30	40 (14.4)	9 (3.9)	<0.001
Inappropriate staring or leering that made you feel intimidated	59 (11.5)	27 (18.6)	15 (15.0)	17 (6.3)	<0.001	57 (20.6)	2 (0.9)	<0.001
Sexual gestures, indecent exposure, or inappropriate display of the body	11 (2.1)	4 (2.8)	3 (3.0)	4 (1.5)	0.50	10 (3.6)	1 (0.4)	0.01
Sexually suggestive comments or jokes that made you feel offended	92 (17.9)	42 (29.0)	19 (19.0)	31 (11.5)	<0.001	71 (25.6)	21 (9.1)	<0.001
Sexually explicit pictures, posters, or gifts that made you feel offended	9 (1.7)	4 (2.8)	2 (2.0)	3 (1.1)	0.49	9 (3.2)	0 (0.0)	<0.005
Repeated or inappropriate invitations to go out on dates	12 (2.3)	7 (4.8)	3 (3.0)	2 (0.7)	0.02	11 (4.0)	1 (0.4)	<0.01
Intrusive questions about your private life or physical appearance that made you feel offended	65 (12.6)	34 (23.4)	15 (15.0)	16 (5.9)	<0.001	56 (20.2)	9 (3.9)	<0.001
Inappropriate physical contact	29 (5.6)	14 (9.7)	6 (6.0)	9 (3.3)	0.03	21 (7.6)	8 (3.4)	0.07
Requests or pressure for sex or other sexual acts	6 (1.2)	6 (4.1)	0 (0.0)	0 (0.0)	<0.001	6 (2.2)	0 (0.0)	0.03
Other unwelcome conduct of a sexual nature (excluding online)	13 (2.5)	8 (5.5)	1 (1.0)	4 (1.5)	0.04	9 (3.2)	4 (1.7)	0.40
Online experiences								
Sexually explicit emails or SMS messages	17 (3.3)	7 (4.8)	1 (1.0)	9 (3.3)	0.27	12 (5.2)	5 (1.8)	0.22
Repeated or inappropriate advances on email, social networking websites, or internet chat rooms	15 (2.9)	11 (7.6)	2 (2.0)	2 (0.7)	<0.001	13 (5.6)	2 (0.7)	0.02
Inappropriate commentary, images, or film distributed on some form of social media without the person’s consent	6 (1.2)	2 (1.4)	0 (0.0)	4 (1.5)	0.65	5 (2.2)	1 (0.4)	0.23
Other unwelcome conduct of a sexual nature that occurred online	7 (1.4)	2 (1.4)	1 (1.0)	4 (1.5)	1.00	5 (2.2)	2 (0.7)	0.46
Summary								
Experienced at least one in-person event	160 (31.1)	74 (51.0)	30 (30.0)	56 (20.7)	<0.001	123 (44.4)	37 (15.9)	<0.001
Experienced at least one online event	34 (6.6)	16 (11.0)	3 (3.0)	15 (5.6)	0.03	24 (8.7)	10 (4.3)	0.05
Experienced at least one in-person or online event	173 (33.6)	75 (51.7)	31 (31.0)	67 (24.8)	<0.001	128 (46.2)	45 (19.4)	<0.001

**Figure 1:**
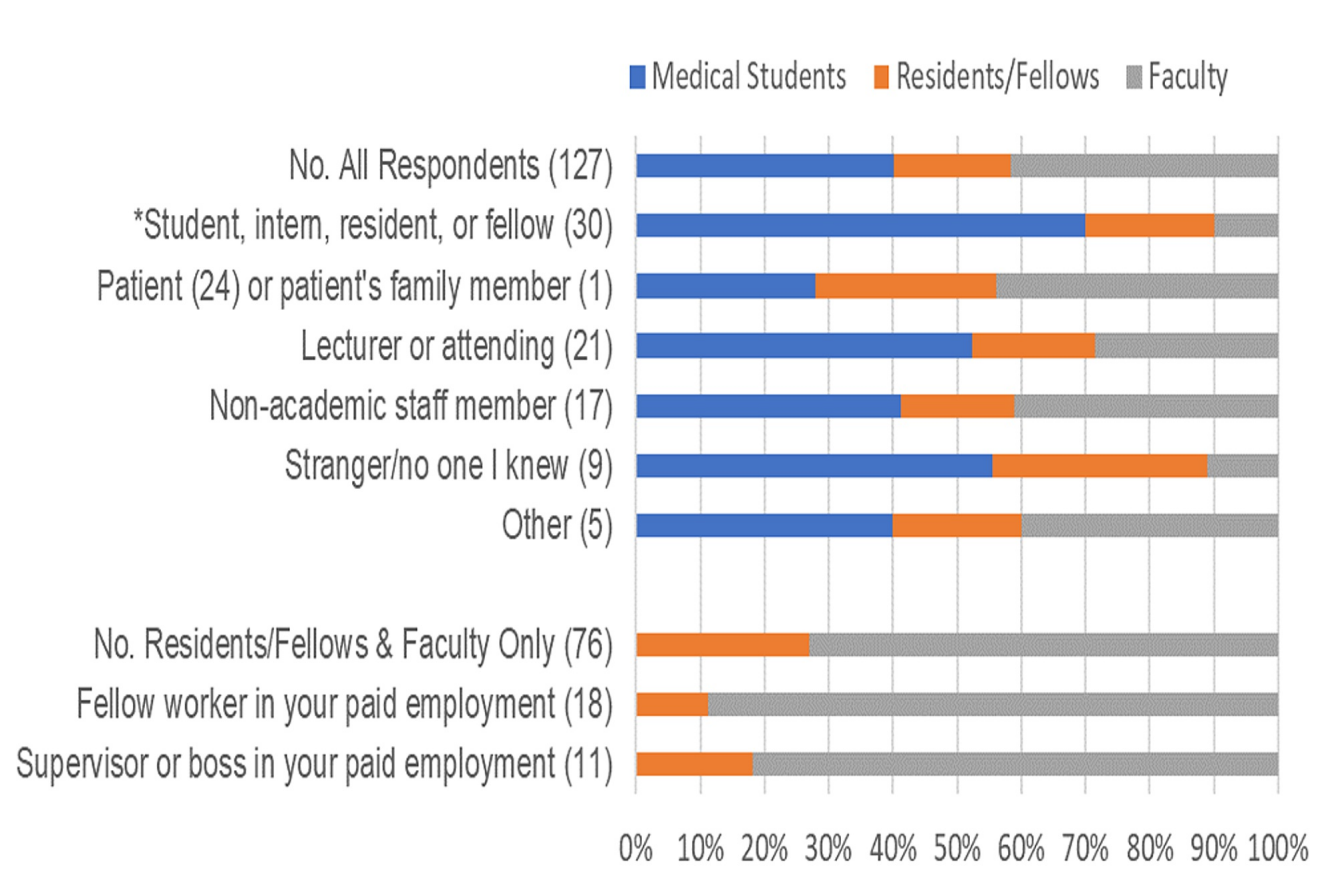
Perpetrators of the most recent sexual harassment experience by status. A total of 127 respondents experiencing sexual harassment identified their perpetrators. Distribution by status is indicated by color for each line. Numbers in parentheses indicate the number of people who classified their perpetrators within that category (e.g., for 30 respondents, the perpetrators were a student, intern, resident, or fellow). Respondents could indicate multiple perpetrators in their most recent experience. As medical students are not employed, two categories of perpetrators are shown separately at the bottom of the figure for residents/fellows and faculty only (n = 76). *p < 0.05

Of the respondents who reported harassment, 73.4% classified the perpetrators (Figure 1). The most common perpetrators were “student, intern, resident, or fellow,” followed by “patient or patient’s family member.” Medical students were most likely to report the perpetrator being a student, intern, resident, or fellow (P < 0.005). There was no difference in patient perpetrators by status.

Participants were asked to describe what the UFCOM/UF Health could do to ensure students, interns, residents, fellows, and faculty know about the university/hospital policies, support services, and reporting processes on sexual harassment. An inductive thematic analysis of the responses to these open-ended questions revealed the following themes:

1) Assuring availability of resources containing the information about policies, support services, and reporting processes: Participants suggested emails with the links to the appropriate information, making sure that a webpage that links to all the required resources is the first hit on online searches, displaying posters/ads with the required information around campus and providing written materials at orientation meetings.

2) Increasing awareness about the subject of sexual harassment: Participants suggested a culture change regarding sexual harassment that should start at the top of the organization, assuring that the UFCOM/UF Health has zero tolerance for this kind of behavior. They suggested that leadership should be more diverse, be open to support those who report someone higher in the hierarchy, and allow for a safe environment for reporting. They expressed that it is important to know there would be no retaliation against the accuser (as long as the facts are truth) such as loss of employment or threats to their careers or status within their departments.

3) Several participants expressed concerns about the current mechanisms for reporting sexual abuse: Participants suggested the process of reporting should be extremely confidential, transparent, standardized, well delineated, and with expected repercussions for the aggressor.

4) General education about the topic of sexual harassment: This ranged from online modules to be completed every 6-12 months to in-person workshops. These training sessions should be tailored to circumstances that apply to the participants, and a clear description of steps to follow should be provided during the training and for reference. In addition, increasing awareness that subtle behavior might be perceived as inappropriate was requested as an addition to current training programs.

Similarly, participants were asked how the UFCOM/UF Health can ensure that students, interns, residents, fellows, and faculty know about the policies, support services, and reporting processes on sexual assault. In general, sexual assault was viewed as a criminal act with the expectation that law enforcement would be involved:

1) Assuring availability and easy access to resources that included all the necessary information.

2) Increasing awareness and a zero-tolerance policy on sexual assault with a hard stance to those found to be guilty of this behavior.

3) Assuring confidentially, transparency, and lack of retaliation to those who come forward and report sexual assault.

4) Continued education and reminders about the topic.

## Discussion

The main findings of this study are that medical students were most likely while faculty were least likely to experience sexual harassment. Overall, 52% of medical student respondents, 31% of resident/fellow respondents, and 25% of faculty respondents experienced sexual harassment in 2018. The most common behaviors were more subtle forms of harassment, that is, sexually suggestive comments or jokes perceived as offensive, followed by intrusive and offensive questions about one’s private life or physical appearance. Notably, only six respondents reported experiencing direct requests or pressure for sex or other sexual acts. All six were medical students. Medical students were significantly more likely to report the perpetrator being a student, intern, resident, or fellow. The pattern whereby medical students experience the most and faculty the least sexual harassment was consistently observed across different types of behaviors.

Our findings suggest that power dynamics inherent to the medical training process are a contributing organizational factor that enable sexual harassment, highlighting a need to further monitor hierarchically dependent relationships. It also serves as a reminder that structural and social forces not only impact our patients’ lives and health but also our own. Indeed, strong departmental hierarchy was previously shown to be the only structural factor significantly associated with sexual harassment among physicians at a German academic medical center, where 70.4% of the respondents reported harassment experiences [7].

A recent study combed through publicly available information on sexual misconduct committed by faculty members and found that 8% of perpetrators were assistant professors, 13% were associate professors, and 51% were professors, suggesting an increasing prevalence by increasing academic tier [8]. Their approach using data available to the public speaks to a public perception that perpetrators of sexual harassment are primarily faculty members. Our results suggest that when sexual harassment behaviors are more comprehensively assessed, the perpetrators are actually varied and include medical students, residents/fellows, faculty members, and patients. Another common public perception is that women are primarily the victims and a male victim is unusual. While our findings do show the expected gender disparity, a sizeable proportion of men also reported being recipients of sexual harassment: 46% of female respondents and 19% of male respondents reported sexual harassment. The silence over males as victims of sexual harassment is another component of the sexual harassment iceberg beneath the surface.

In a report on the sexual harassment of women, the NASEM likened sexual harassment to an iceberg. The public perception of sexual harassment is akin to an iceberg, where the most egregious behaviors lie above the water but the bulk of sexual harassment behaviors lie beneath the surface of public consciousness. The highest tip of the iceberg represents sexual coercion (e.g., quid pro quo for sexual favors), which sits atop unwanted sexual attention (e.g., rape, sexual assault, unwanted groping/stroking). Sitting below sea level and below public consciousness, often remaining unseen, are behaviors (e.g., sexual insults, offensive remarks, and pressure for sexual favors) that tend to be absent from public consciousness [2], and consequently, from surveys of sexual harassment in academic medicine.

In 2006, the “Me Too” movement was founded to help survivors of sexual violence, specifically minorities and women from low socio-economic backgrounds. Within six months, because of the twitter hashtag #metoo, this movement became viral and was propelled into a national dialogue [9]. In response to the “Me Too” movement, the “Times Up” movement was founded in 2018 by Hollywood celebrities with the goal of providing safety, equity, and power to women in the workplace. As part of the Times Up Healthcare movement, healthcare organizations across the country were invited to make a commitment to end gender inequity and sexual harassment [10]. In 2019, the University of Florida joined the Times Up Healthcare Consortium pledging to prevent sexual harassment and gender inequity, and to protect and aid those who are targets of harassment and discrimination. Further, the results from this study are to be used at the institution to advance the mission of preventing harassment and discrimination.

Limitations include the possibility of non-response bias. Sexual harassment surveys often have low response rates secondary to fear of retaliation despite assured confidentiality and unwillingness to share experiences because of emotional repercussions [2]. Although our response rate was nearly twice that of the original survey instrument [6], it remains difficult to discern whether our estimates are inflated (experiences motivating responses) or deflated (stigma/fear decreasing responses to a single-institution survey). To mitigate this bias, the invitation explicitly stated appreciation of participation regardless of personal experiences. It is also unknown if non-response bias varies by hierarchical status. However, if such bias existed, the expected impact would be that those with less power underreport and those with more power overreport, which would only strengthen our finding.

## Conclusions

Medical students were most likely to experience sexual harassment, followed by residents and fellows and faculty. These findings suggest that sexual harassment incidence tends to decrease with increasing academic hierarchy. Our study’s inclusion of medical students and distinction between resident/fellow and faculty physicians further highlights the insidious effects of the hierarchy and the need to diffuse hierarchically dependent relationships. The three most common types of sexual harassment behavior in our study fell below public consciousness, suggesting that many estimates currently in the literature may actually be gross underestimates. Academic institutions must seek and identify their own unique cultures of sexual harassment to address the problem and enact change.
